# 3D-Printed Hydrogels as Photothermal Actuators

**DOI:** 10.3390/polym16142032

**Published:** 2024-07-17

**Authors:** Melanie M. Ghelardini, Martin Geisler, Niclas Weigel, Jameson P. Hankwitz, Nicolas Hauck, Jonas Schubert, Andreas Fery, Joseph B. Tracy, Julian Thiele

**Affiliations:** 1 Department of Materials Science and Engineering, North Carolina State University, Raleigh, NC 27695, USA; mmghelar@ncsu.edu (M.M.G.);; 2 Leibniz Institute of Polymer Research Dresden, Institute of Physical Chemistry and Polymer Physics, 01069 Dresden, Germany; geisler@ipfdd.de (M.G.);; 3 Institute of Physical Chemistry and Polymer Physics, Technische Universität Dresden, 01062 Dresden, Germany; 4 Institute of Chemistry, Otto von Guericke University Magdeburg, Universitätsplatz 2, 39106 Magdeburg, Germany

**Keywords:** 3D printing, bioplotting, poly(*N*-isopropylacrylamide), gold nanorods, cycloaddition, gelatin, photothermal heating

## Abstract

Thermoresponsive hydrogels were 3D-printed with embedded gold nanorods (GNRs), which enable shape change through photothermal heating. GNRs were functionalized with bovine serum albumin and mixed with a photosensitizer and poly(*N*-isopropylacrylamide) (PNIPAAm) macromer, forming an ink for 3D printing by direct ink writing. A macromer-based approach was chosen to provide good microstructural homogeneity and optical transparency of the unloaded hydrogel in its swollen state. The ink was printed into an acetylated gelatin hydrogel support matrix to prevent the spreading of the low-viscosity ink and provide mechanical stability during printing and concurrent photocrosslinking. Acetylated gelatin hydrogel was introduced because it allows for melting and removal of the support structure below the transition temperature of the crosslinked PNIPAAm structure. Convective and photothermal heating were compared, which both triggered the phase transition of PNIPAAm and induced reversible shrinkage of the hydrogel–GNR composite for a range of GNR loadings. During reswelling after photothermal heating, some structures formed an internally buckled state, where minor mechanical agitation recovered the unbuckled structure. The BSA-GNRs did not leach out of the structure during multiple cycles of shrinkage and reswelling. This work demonstrates the promise of 3D-printed, photoresponsive structures as hydrogel actuators.

## 1. Introduction

Hydrogel-based functional systems have potential for biomedical and related applications in soft and living systems, for instance in tissue engineering, soft actuators or robots, and sensing applications [1,2,3]. In these systems, untethered or remote stimulation is appealing for both actuation within confined spaces and in vivo medical devices. Photothermal triggering of thermoresponsive hydrogels with embedded pigments is a well-known concept for remote stimulation, where plasmonic Au nanoparticles (NPs) are commonly used for their chemical inertness and high extinction coefficients, which make them efficient for photothermal heating [4,5,6]. Gold nanorods (GNRs) provide further tunability of the extinction into the near infrared (NIR) spectrum, which is essential for deeper penetration through blood and tissues [7,8]. While photothermal heating of thermoresponsive hydrogels has great potential for remotely actuated hydrogel robots, the ability to 3D print these structures would enable fabrication of more complex structures and rapid prototyping [9,10,11,12].

Poly(*N*-isopropylacrylamide) (PNIPAAm) is a thermoresponsive hydrogel with a characteristic lower critical solution temperature (LCST) that is slightly above room temperature, at which the hydrogel transitions from a swollen, transparent state into a shrunken, phase-separated, and opaque state [13,14,15]. While there are numerous examples of photothermal triggering of PNIPAAm using embedded Au NPs [4,5,6] and GNRs [16,17,18,19], their application as actuators is generally limited to simple designs that can be fabricated in molds or through photolithography, because the rheology of unmodified PNIPAAm precursor solutions is often too liquid-like for many 3D printing methods [20]. Although 3D printing of hydrogels is a rapidly growing field, much work is still needed to establish and optimize relationships among ink chemistry and processing parameters to design inks with the appropriate rheology, as well as to analyze the structure and properties of the 3D-printed objects [21,22].

Here, we report an investigation that combines thermoresponsive hydrogels, 3D printing, and plasmonic NPs for photothermal heating and remote stimulation of free-standing 3D-printed structures. PNIPAAm-GNR composites are 3D printed using embedded direct ink writing (DIW) of inks containing both photocrosslinkable PNIPAAm hydrogel precursors and dispersed GNRs. The inks are deposited into a sacrificial support matrix composed of an acetylated gelatin hydrogel and then photocrosslinked with ultraviolet (UV) light to form the hydrogel in situ. After removing the acetylated gelatin support, NIR illumination of the structures with a light emitting diode (LED) drives photothermal heating and shrinkage of the PNIPAAm, which has potential applications in hydrogel soft robotics. In the following sections, we introduce relevant prior studies that have inspired and enabled this work.

The primary approaches for 3D printing PNIPAAm are extrusion-based printing, e.g., based on pneumatic actuation, where PNIPAAm is mixed with a rheology modifier to increase the ink viscosity, and lithography-based techniques, including digital light processing (DLP) and multiphoton lithography [18,20,23,24,25,26]. An advantage of DIW over DLP is elimination of the photoabsorber, e.g., Sudan I, which is an important component of the resin formulation, but has an overlapping extinction with GNRs. Incorporating GNRs in DLP would therefore interfere with control over polymerization and vertical print resolution. In the DIW approach used in this work, GNRs also interfere with photopolymerization because they absorb UV light, but use of a sacrificial support matrix substantially decouples the timing of deposition of the ink and photopolymerization. We hypothesize that this will make the process more tolerant to differences in the ink than DLP, such as the viscosity and rate of crosslinking, as well as in the loading of GNRs. The support matrix additionally relaxes the rheology requirements for DIW and enables use of less viscous inks [27]. An ideal sacrificial support matrix is one that is easily removed, can provide a high-resolution print, and is biocompatible [28]. Many materials exhibit these properties and have been used as sacrificial supports, including carbohydrate glass [29], xanthan gum [30], and gelatin [31,32,33]. Alternatively, modifications can be made to the ink, compensating for the rheological limitations and liquid-like nature of PNIPAAm precursors and endowing them with the required shear thinning behavior for extrusion-based printing. Demonstrating this, DIW of PNIPAAm has been reported by mixing with polyvinyl alcohols [34], polyurethanes [25], nanoclays [24,35], alginate [23], or gel-phase Pluronic F127, which can be dissolved later [36].

Incorporating GNRs in 3D-printed hydrogels by adding them to the ink enables remote photothermal heating and actuation of thermoresponsive hydrogel structures. Light absorbed by GNRs drives non-radiative relaxation processes, generating heat that is then transported into the surroundings [7]. The rod shape splits the surface plasmon resonance into the longitudinal surface plasmon resonance (LSPR) and transverse surface plasmon resonance (TSPR). The LSPR corresponds to oscillations of conduction electrons along the long axis of the GNR, whereas the TSPR corresponds to oscillations along a short axis. The LSPR is of interest for this work because it is more intense than the TSPR and can be tuned by adjusting the aspect ratio of the GNRs [37]. Photothermal heating of plasmonic NPs has been investigated for numerous applications, including photothermal therapy and triggered drug release [7,8,38,39,40], catalysis [41,42,43], steam generation [44,45,46,47], and triggering responsive polymers [48,49], which includes the deswelling of PNIPAAm structures [4,16], PNIPAAm coatings on NPs [6,50], and hydrogel-based soft robotics [17,18,51]. To the best of our knowledge, 3D printing of free-standing PNIPAAm structures with GNRs for photothermal heating has not been reported previously, but there has been a recent report of 3D-printed bilayer structures that bend through interfacial stresses [26]. Graphene oxide has also been used for photothermal heating of PNIPAAm structures but lacks the tunable extinction of GNRs [52].

## 2. Materials and Methods

### 2.1. Gold Nanorod Synthesis and Functionalization

GNRs were synthesized according to an established method that yields 1 L of solution containing ≈190 mg of GNRs stabilized by cetyltrimethylammonium bromide (CTAB-GNRs) with dimensions of 81 × 23 nm [53], which were then functionalized with bovine serum albumin (BSA) [54]. The CTAB-GNRs were purified by first removing excess CTAB through centrifugation to reduce the concentration to ≈0.9 mM, near the critical micelle concentration of CTAB. A total of 50 mL of the as-synthesized CTAB-GNRs, which contain 0.1 M CTAB, was centrifuged (Sorvall Legend X1R with Fiberlite F15-6x100y rotor, Thermo Fisher Scientific, Waltham, MA, USA) at 11,000 rpm (13,280× *g*) for 20 min, and as much of the supernatant was removed as possible without perturbing the pellet of sedimented CTAB-GNRs. The total volume was increased to 50 mL by adding deionized water (ACS Reagent grade, ASTM Type I, ASTM Type II, Ricca, Arlington, TX, USA), followed by gently sonicating to redisperse the CTAB-GNRs and completing a second round of centrifugation. After removing the supernatant as described above, the CTAB-GNRs were redispersed in 50 mL of 0.9 mM CTAB (Amresco, high purity, Albany, NY, USA) in deionized water and centrifuged a third time. Upon removing the supernatant, the CTAB-GNRs were redispersed in 25 mL of 0.9 mM CTAB. Immediately afterward, the GNR solution was added to a 50 mL aqueous solution of 10 mg mL^−1^ BSA (Sigma-Aldrich, A7906, 98%, St. Louis, MO, USA) and 0.02% (*w*/*w*) sodium (Na) citrate dihydrate (Mallinckrodt, 99% min, St. Louis, MO, USA) while sonicating this BSA solution. The mixture was sonicated for 30 min, while maintaining the temperature of the water bath below 35 °C by periodically adding ice to the bath.

The product was then divided into three 25 mL aliquots, which were centrifuged at 7000 rpm (5380× *g*) for 20 min. After centrifugation, a second round of functionalization with BSA was performed to maximize the extent of BSA functionalization on the GNRs. Pellets from the three centrifuge tubes were combined and brought to a total volume of 50 mL with a pH-adjusted, aqueous, 0.02% (*w*/*w*) Na citrate solution of lower BSA concentration, 1 mg mL^−1^. The pH was adjusted by adding 100 µL of 0.1 M NaOH (Sigma-Aldrich, 98%, St. Louis, MO, USA) per 10 mL of solution, resulting in a pH of ≈10.9. The mixture was allowed to react for 24 h at room temperature without agitation. After completing the reaction, purification was performed by dividing the 50 mL solution between two centrifuge tubes and completing three rounds of centrifugation at 7000 rpm for 20 min using the deionized water (adjusted to pH ≈10.9 with NaOH) as the solvent, yielding BSA-GNRs. The pellets from the two centrifuge tubes were combined and dispersed in a total of 50 mL of the same pH-adjusted water.

### 2.2. Preparation of Hydrogel Macromer

Thioxanthone (TXS) photosensitizer and 2-(dimethyl-maleimido)-*N*-ethyl-acrylamide (DMMIAAm) monomer were synthesized and characterized according to an established procedure [55]. The PNIPAAm macromer was synthesized by modifying a procedure for the free-radical polymerization of *N*-isopropylacrylamide (NIPAAm, 99%, Acros Organics, Geel, Belgium) and DMMIAAm in the presence of azobisisobutyronitrile (AIBN, Sigma-Aldrich, St. Louis, MO, USA) [56]. NIPAAm was recrystallized twice from dry hexane (>98.5%, Emparta, Merck KGaA, Darmstadt, Germany). AIBN was recrystallized from cold MeOH (99.9%, for analysis, Acros Organics, Geel, Belgium). Then, 3.411 g of NIPAAm, 355.3 mg of DMMIAAm, and 34.6 mg of AIBN were dissolved in 17 mL of anhydrous *N,N*-dimethylformamide (DMF, 99.8%, extra dry, Acros Organics, Geel, Belgium). The mixture was stirred under argon atmosphere for 24 h at 70 °C. The PNIPAAm macromer was precipitated by adding the reaction mixture dropwise to 400 mL of diethyl ether (DEE, Chemsolute, 99.5%, Th. Geyer GmbH & Co. KG, Renningen, Germany). After removal of DEE by vacuum filtration, the precipitate was dissolved in a few mL of chloroform (>99%, dried over molecular sieve, Acros Organics, Geel, Belgium) and precipitated in DEE three more times to ensure the complete removal of DMF and NIPAAm monomer. Subsequently, the polymer was dried at 10^−2^ bar. The acrylamide-2-(dimethyl-maleimide (DMMI-acrylamide) fraction in the copolymer was calculated to be 4.5 mol% from ^1^H-NMR spectroscopy (CDCl_3_) by comparing integrated values of the peaks assigned to the ethyl protons of DMMI-acrylamide (-CH_2_-CH_2_-, 3.10–3.75 ppm) and the proton of the tertiary carbon of the isopropyl group (-CH-(CH_3_)_2_, 4.01 ppm). Size-exclusion chromatography measurements of the PNIPAAm macromer were performed on an Agilent LC 1100 utilizing a PLgel MIXED-C column (Agilent Technologies GmbH, Waldbronn, Germany) with an eluent composed of dimethylacetamide (DMAc), 2% (*v*/*v*) water, and 3 g L^−1^ lithium chloride operated at a flow rate of 0.8 mL min^−1^. Samples were dissolved at a concentration of 2 mg mL^−1^ and passed through a 0.2 μm syringe filter. Calibration was performed with poly(2-vinylpyridine) (P2VP) standards. Hence, molecular weights were obtained as apparent P2VP equivalent values.

Yield: 2.155 g. ^1^H-NMR (500 MHz, CDCl_3_): d = 6.61 (br, -NH-), d = 4.01 (s,-CH-(CH_3_)_2_), 3.10–3.75 (br,-CH_2_-CH_2_-), 2.40–1.30 (br, backbone + DMMI CH_3_ protons), 1.16 (s, -CH-(CH_3_)_2_). SEC [Poly(2-vinylpyridine) standard]: *M*_n_ = 32,000 g mol^−1^, *M*_w_ = 108,000 g mol^−1^, *M*_w_/*M*_n_ = 3.38.

### 2.3. Preparation of PNIPAAm/GNR Composite Ink for 3D Printing

The base ink formulation was the same for all samples, irrespective of the loading of BSA-GNRs. A total of 500 µL of either ultrapure water (from a Millipore Milli-Q Direct 8 water purification system, Burlington, MA, USA) or BSA-GNR solution was added to 37.5 mg of PNIPAAm macromer. Inks were prepared over a range of loadings with BSA-GNRs by controlling the extinction or optical density (OD) at the peak LSPR extinction of the BSA-GNR solution. The BSA-GNR solutions were prepared such that they had an OD of 2.5, 5, 10 or 20 for BSA-GNRs, when measured (via quantitative dilution) in a cuvette with path length of 1 cm. These concentrations were obtained through quantitative dilution of a stock solution of BSA-GNRs or concentration by centrifugation. The concentration-adjusted BSA-GNRs (or ultrapure water, for the unloaded control) were added to the PNIPAAm macromer powder, followed by vortexing and mild sonication in an ice bath to dissolve the macromer, while maintaining the temperature well below the LCST to avoid phase separation of the PNIPAAm macromer. A volume of 10 µL of 0.1 M TXS photosensitizer was then added to drive crosslinking during 3D printing. After additional gentle sonication, the ink was used immediately for 3D printing.

### 2.4. Preparation of Acetylated Gelatin Microgel Sacrificial Support Matrix

The material for the sacrificial support matrix was prepared on a 3–5 g scale by modifying a procedure for acetylated gelatin [57]. Gelatin (from porcine skin, gel strength 300 g Bloom, Sigma-Aldrich, St. Louis, MO, USA) was dissolved by stirring at a concentration of 5% (*w*/*w*) in ultrapure water at 60 °C for 30 min. For each gram of gelatin, 2 mL of acetic anhydride (ReagentPlus, ≥99%, Sigma-Aldrich, St. Louis, MO, USA) and 300 µL of a 60 g L^−1^ solution of 4-dimethylaminopyridine (>99%, TCI Chemicals, Eschborn, Germany) in pyridine (anhydrous, 99.8%, Sigma-Aldrich, St. Louis, MO, USA) were added to the mixture and stirred at 60 °C for 80 min. The solution was then transferred into a dialysis bag (MWCO 10 kDa, ThermoFisher Scientific Inc., Waltham, MA, USA) and dialyzed against ultrapure water for 2 days. A pale colorless solid product was obtained by rotary evaporation. Partial acetylation was confirmed by ^1^H NMR [58] using a Bruker Avance III 500 with D_2_O (Sigma-Aldrich, St. Louis, MO, USA) as the solvent and by confirming a reduced melting point (<28 °C) of the 5% (*w*/*w*) acetylated gelatin hydrogel prepared with ultrapure water.

Pieces of 5% (*w*/*w*) acetylated gelatin hydrogel were prepared in ultrapure water by dissolution under stirring for 30 min at 60 °C and then cooling overnight at 4 °C. A total of 100 mL of ice-cold ultrapure water per 50 mL of 5% (*w*/*w*) acetylated gelatin was then added, and the hydrogel was ground into a fine particle slurry using a T25 Ultra-Turrax grinder (IKA-Werke GmbH & Co. KG, Staufen im Breisgau, Germany) at 16,000 rpm for 3 min. The resulting microgel slurry was collected by centrifugation at 8000 rpm (6650× *g*) using a Sigma 3K30 centrifuge (Sigma Laborzentrifugen GmbH, Osterode am Harz, Germany), and the supernatant was discarded. This slurry, which serves as the support matrix, was stored at 4 °C until further use.

### 2.5. Printing Process and Removal of Acetylated Gelatin Microgel Support Matrix

Aluminum foil was shaped into a small bath for the acetylated gelatin microgel support matrix by first wrapping it around a small container (2.5 cm long and wide, 1 cm tall) with an open bottom as a template. The template was removed, and a hole was cut in one side of the aluminum foil container to enable UV illumination from the side. The aluminum foil container was placed on a glass slide and then filled with the support matrix slurry. The hole for UV illumination did not affect the mechanical integrity of the support matrix. The same aluminum foil container was reused for all prints. The PNIPAAm/GNR ink was printed using a 3D-Bioplotter system (Manufacturer Series, EnvisionTEC GmbH, Gladbeck, Germany) in combination with a LumenDynamics OmniCure S1500 UV light source (Excelitas, Pittsburgh, PA, USA) with a wavelength range of 240–450 nm used for curing. The adjustable shutter on the source was set to 35%, which corresponds to an output power of 4.17 W cm^−2^. A standard crosshatch structure with dimensions of 13 mm × 13 mm was printed over 25 layers to achieve a total height of 3.6 mm, as shown in Figure S11. 

A total of 500 µL of PNIPAAm/GNR ink was transferred into a disposable cartridge for pneumatic extrusion equipped with a 30-gauge needle (150 µm inner diameter). To compensate for the small volume of ink filled into the cartridge, paraffin oil was added to create a flat, level surface to ensure uniform application of pressure during 3D printing. The needle was inserted directly into the acetylated gelatin support matrix, and the structure was continuously printed within it at a constant pressure of 0.2 bar and a print speed of 28 mm s^−1^. Between layers, the needle was removed and then reinserted into the support matrix at the new height. The support matrix and 3D-printed structure were illuminated with UV light throughout the printing process. A secondary curing step was performed after printing and before removing the aluminum foil container to drive further crosslinking, where the UV light illuminated the sample from above and was manually tilted at different angles and moved around all sides of the sample for 3 min. To mitigate undesired heating during 3D printing and maintain temperatures below the LCST of PNIPAAm, the print head’s built-in Peltier cooling was to set to 20 °C, while the build platform’s thermoliquid-supplied cooling was set to 2 °C. 

After 3D printing, the sample was refrigerated at 4 °C with the structure still encased in the acetylated gelatin microgel until further use. To extract the 3D-printed hydrogel structure, the aluminum foil container was carefully lifted from the support matrix block, and the acetylated gelatin support structure was removed by submerging the glass slide with the support matrix and embedded structure in a 1 L ultrapure water bath at 29 °C with gentle stirring by hand. At this temperature, the acetylated gelatin melts and dissolves into the water, leaving the 3D-printed structure submerged in the water bath.

### 2.6. Characterization of GNRs

The GNRs were imaged using a ThermoFisher Talos F200x (Waltham, MA, USA) transmission electron microscope operated at 200 kV. The average dimensions were obtained from measurements of 200 GNRs. Optical extinction spectra were acquired with an Ocean Optics CHEMUSB4-VIS-NIR spectrophotometer (Orlando, FL, USA) equipped with a glass cuvette with a path length of 1 cm. Samples were prepared by diluting 0.5 mL aliquots to 3.0 mL using deionized water with dissolved CTAB or NaOH at a concentration to match that of the sample and prevent unintentional agglomeration. A Malvern Zetasizer Nano (Westborough, MA, USA) was used for ζ-potential measurements.

### 2.7. Characterization of 3D-Printed Materials

A Leica TCS SP8 laser scanning confocal microscope with a 40× objective lens and operating in tile-scan mode was used for image fluorescence with 488 nm laser excitation from a sample without GNRs and loaded with 1 g L^−1^ fluorescein isothiocyanate-dextran (2 MDa, Sigma-Aldrich, St. Louis, MO, USA). Optical brightfield microscopy was performed with a Leica DMi8 microscope. A cross-section of an OD 5 sample that had been flash-frozen in liquid nitrogen was imaged with a Zeiss Neon 40 EsB scanning electron microscope (Carl Zeiss Microscopy GmbH, Jena, Germany). Compression testing was performed using a uniaxial Zwick 1456 testing machine with a load cell of 5 N (ZwickRoell GmbH & Co. KG, Ulm, Germany). The strain rate was 1 mm min^−1^, and the temperature was maintained at 25 °C. Samples were prepared for compression testing by bulk polymerization in a cylindrical mold (5 mm diameter and 4 mm high) with crosslinking using the same UV light and exposure conditions to mimic the 3D printing process.

### 2.8. Convective Heating

The 3D-printed structures were equilibrated in an ultrapure water bath at room temperature. After equilibration, a video recording of the actuation process was started. The water bath was rapidly heated on a hot plate to 36–37 °C, which is above the LCST of PNIPAAm. This temperature was reached within 8 min. The heat supply was then switched off, and ice (from ultrapure water) was added to promote reswelling and a return to equilibrium before photothermal heating experiments. A photograph of the setup for convective heating is shown in Figure S12. The area of the structure in different states was measured as the contour of its outer boundary using the polygon tool in ImageJ 1.53k. For each cycle, the dimensions of the most swollen state were measured. Snapshots of the most shrunken state were taken at the maximum temperature before beginning the cooling cycle.

### 2.9. Photothermal Heating

The 3D-printed structures were placed in a glass Petri dish filled with ultrapure water and 13 mm away from the LED with a peak emission at 850 nm (Osram LZ4-00R608 assembled in an aluminum housing and with a lens to spread the light, Hillsboro, NH, USA). A thermal imaging camera (Seek Thermal Compact Pro, Santa Barbara, CA, USA) was added to the setup. The LED was switched on at its highest intensity while recording videos and performing thermal imaging. After the structure heated past the LCST of PNIPAAm and a quasi-steady state was reached, the LED was turned off. Snapshots from the video before turning on the LED and immediately after switching it off were used for analysis of the swollen and shrunken states, respectively. Photographs in Figure S13 show the setup for photothermal heating. Since the temperature of the water bath remained below the LCST, the print reswelled as heat was transported away, and its temperature dropped below the LCST. After equilibration for at least 10 min after turning off the LED, the next cycle of photothermal heating was started by turning the LED back on. Three successive cycles of photothermal heating were performed with each sample.

## 3. Results and Discussion

A custom-synthesized PNIPAAm precursor with statistically incorporated UV-crosslinkable groups was chosen to facilitate crosslinking via polymer-analogous photogelation (PAG) based on a [2 + 2] cycloaddition mechanism at room temperature. We refer to this precursor as a macromer because of the prepolymerization step, where the additional incorporation of reactive 2-(dimethyl-maleimido)-*N*-ethyl-acrylamide (DMMIAAm) units enables crosslinking and hydrogel network formation. In PAG, using prepolymerized macromers separates the crosslinking and polymerization steps, which is known to provide improved control and increased nanoscale and macroscale homogeneity of the resulting hydrogels in comparison with free-radical crosslinking of monomers [56,59]. The resulting hydrogels can have defined concentrations of specific functional groups, wherein a homogeneous, physically entangled pre-gel structure is finalized through [2 + 2] cycloaddition [60].

**Figure 1:**
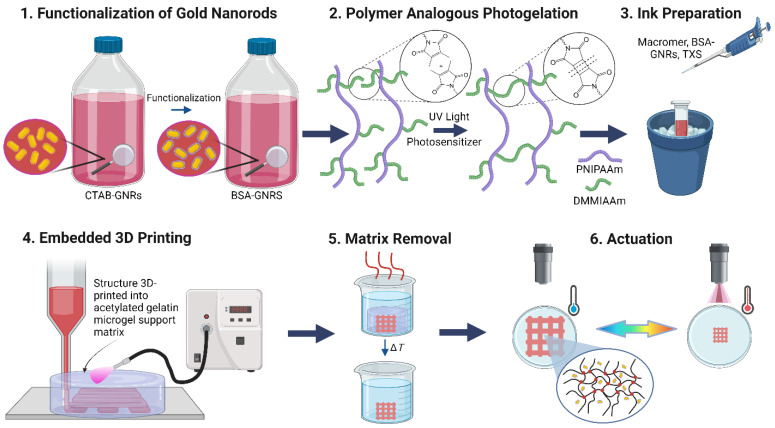
Schematic overview of material design and experimental approach for fabrication and actuation.

A significant advantage of PAG in this work is that crosslinking is performed at room temperature and yields transparent structures [56,61], in contrast to the exothermic nature of free-radical polymerization, which exacerbates morphological inhomogeneities that result in optical scattering and opacity. Furthermore, crosslinking of the macromer below its LCST in PAG allows for 3D printing of objects in the swollen state rather than shrunken state, which improves their fidelity with the designed structure. GNRs were coated with bovine serum albumin (BSA-GNRs) and then loaded into PNIPAAm macromer inks at different concentrations and printed into a sacrificial support matrix composed of an acetylated gelatin microgel slurry with simultaneous crosslinking via PAG. The BSA-GNRs remained well dispersed within the printed structure. The as-designed structures were 13 mm × 13 mm, with crosshatched squares continuously printed over 25 layers to achieve a height of 3.6 mm. After 3D printing and curing, the acetylated gelatin was removed through gentle heating in a water bath, and the structure was actuated through convective heating, or photothermal heating with NIR light. A similar response was observed for all loadings of GNRs and for both types of actuation, with all samples shrinking to approximately 20–30% of their initial area. An overview of the experiments is presented in Figure 1.

### 3.1. Preparation of Ink and Sacrificial Support Matrix

**Figure 2:**
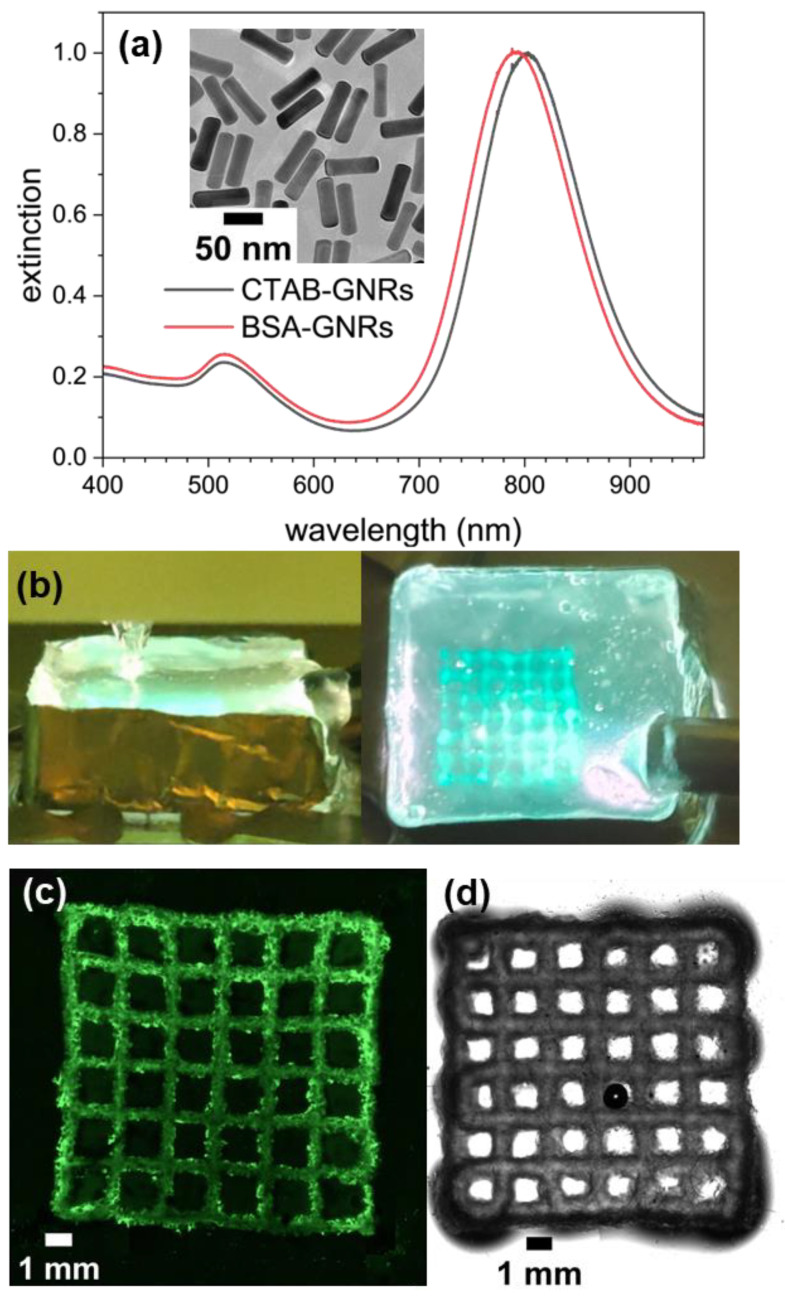
(**a**) Extinction spectra of aqueous CTAB-GNRs and BSA-GNRs normalized to peak extinction of 1 with an inset TEM image of BSA-GNRs; (**b**) DIW within a sacrificial support matrix of acetylated gelatin under UV exposure; (**c**) confocal fluorescence microscopy image of an unloaded structure dyed with fluorescein; (**d**) bright-field microscopy image of a GNR-loaded structure (OD 10), where the black dot in the center is an air bubble.

A PNIPAAm macromer with a number-average molecular weight of 32 ± 2 kg mol^−1^ and a molar mass dispersity of 3.4 ± 0.1 was synthesized according to established methods [55,56]. DMMIAAm was incorporated as a co-monomer at 4.5 mol% in the macromer synthesis. Further incorporation of DMMIAAm would increase the hydrophobicity, resulting in a decreased LCST and swelling ratio [59,62,63]. While more crosslinking could result in a tougher hydrogel, a lower extent of crosslinking was chosen, thus giving a high swelling ratio and retaining the native LCST to show the potential of these materials and structures for photothermal heating and remote actuation. For future applications, where mechanical robustness is preferred over swelling changes, increasing the DMMIAAm content could be investigated. The incorporation and concentration of DMMIAAm groups was verified and quantified by NMR (Figure S1). GNRs stabilized by cetyltrimethylammonium bromide (CTAB-GNRs) with average dimensions of 81 × 23 nm and an LSPR maximum at 802 nm were synthesized according to a known procedure (Figure 2a, Table S1) [53]. The native CTAB coating was replaced with BSA using a modified procedure [54] to mitigate known challenges with CTAB coatings. Specifically, excess CTAB is required to maintain stable, well dispersed CTAB-GNRs, which are also susceptible to agglomeration when mixing with other species. BSA coatings address these limitations, improve the colloidal stability of GNRs, and are biocompatible [54]. BSA has also been used previously together with PNIPAAm grafted to the surfaces of Au NPs to create multiresponsive coatings [6]. The small blueshift in the LSPR peak is consistent with a change in the dielectric environment on the surface of the GNR when replacing CTAB with BSA, while maintenance of a narrow breadth of the peak indicates the BSA-GNRs are well dispersed and not agglomerated [54]. The change from positive to negative ζ-potential (Table S1) is also consistent with replacing CTAB with BSA [64]. After mixing PNIPAAm macromer and BSA-GNRs, thioxanthone disulfonate (TXS) was added as a photosensitizer [65], which was synthesized according to an established method [55].

Aqueous inks containing BSA-GNRs, PNIPAAm macromer (6.85% *w*/*w*), and TXS (1.83% *w*/*w*) were prepared for DIW. BSA-GNRs were omitted from the control sample. The low-viscosity inks require a sacrificial support matrix to preserve the structure during printing and curing. In this work, a sacrificial support matrix composed of acetylated gelatin [57] was introduced. Acetylation reduces the melting temperature of gelatin [66] below the LCST of PNIPAAm, which facilitates removal of the support while maintaining the 3D-printed PNIPAAm structure in its swollen state. In comparison, reliably removing gelatin support matrices requires heating up to 37 °C, which is above the LCST [31,32]. Acetylated gelatin was prepared by treating dissolved gelatin with acetic anhydride and 4-dimethylaminopyridine [57]. Acetylation was confirmed by NMR (Figure S2). The product was purified by dialysis, and as-prepared 5% (*w*/*w*) macroscopic pieces of acetylated gelatin were ground into microgel particles. The microgel was concentrated by centrifugation into a fine, transparent slurry, which formed a self-supporting mound when transferred onto a glass slide for use as a sacrificial support matrix. An aluminum foil box was placed around the mound to provide mechanical reinforcement and to reflect illumination back into the matrix (Figure 2b). As the needle moved through the matrix and ink was deposited, the acetylated gelatin microgel matrix behaved as a viscous liquid that was displaced and then self-healed, preventing deformation and spreading of the deposited ink.

### 3.2. Embedded 3D Printing and Characterization

During embedded 3D printing and photocrosslinking of a crosshatched structure, the needle moved into the support matrix, and each layer of the structure was printed continuously. Throughout the entire 2 min printing process, the UV light source was switched on, illuminating the build platform from the side, as shown in Figure 2b and Movie S1. After depositing the ink, the UV light was moved over the top surface of the support matrix with the encased print as well as around all four sides for a total post-cure time of 3 min to maximize crosslinking throughout the hydrogel. The print head was cooled to 20 °C by thermoelectric cooling, while the print stage was cooled to 2 °C via thermoliquid-supplied cooling. Active cooling of the support matrix compensated for heat evolution from the UV source and scattered UV light during printing and maintained the temperature below the LCST. Even though the maximum extinction of the GNRs was at NIR wavelengths, the extinction in the blue and UV was also significant and could potentially induce photothermal heating of the deposited PNIPAAm. After 3D printing and post-curing, the support matrix with the embedded print was placed in a water bath at 29 °C, melting the acetylated gelatin and leaving behind only the PNIPAAm structure (Movie S2). Images from confocal fluorescence microscopy of an unloaded 3D-printed structure dyed with fluorescein and bright-field microscopy of a structure loaded with BSA-GNRs (Figure 2c,d) provide clear views of the crosshatches.

**Figure 3:**
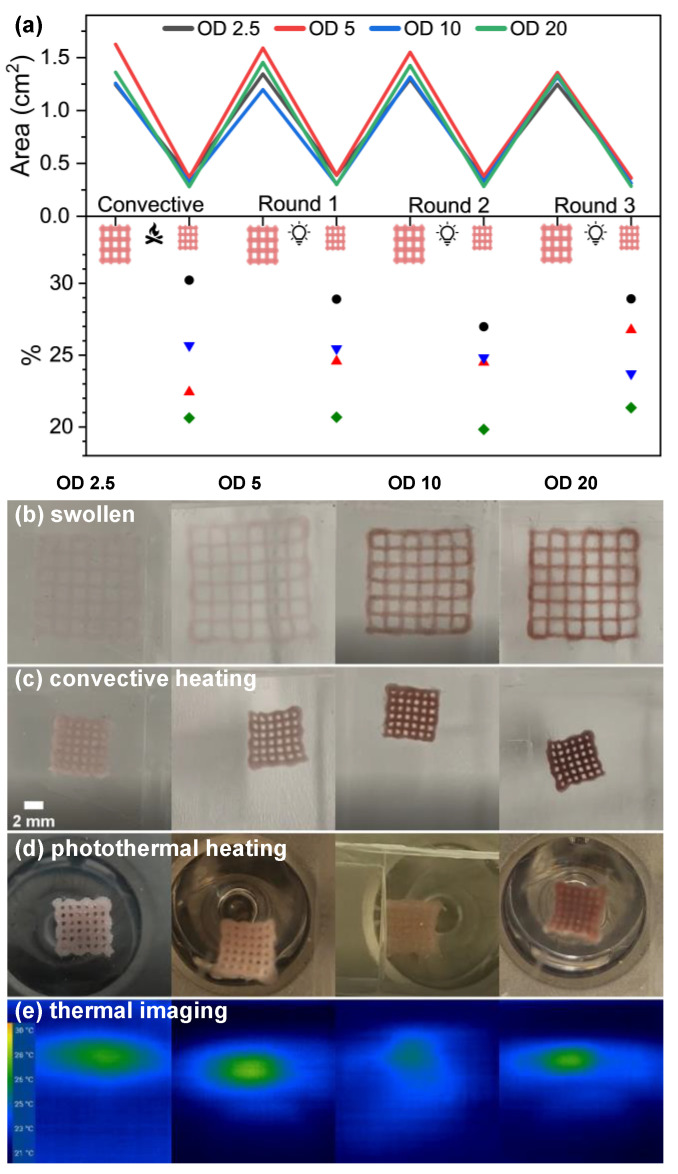
Shrinkage and reswelling behavior during one cycle of convective heating followed by three cycles of photothermal heating for OD 2.5, 5, 10, and 20 samples loaded with GNRs. (**a**) Measurements of the area during cycling in the top panel and the area of the shrunken structure divided by the area of the swollen structure in the bottom panel highlight minor differences in the shrinkage behaviors. To assess the error in the measurements, 10 measurements were taken of the area of an OD 10 sample in the swollen and shrunken state during the second cycle of photothermal heating, from which respective standard deviations of 0.019 and 0.0039 cm^2^ were calculated. These errors propagated into an error of the ratio of the shrunken/swollen areas of 0.45% for the second cycle of photothermal heating for the OD 10 sample. These errors are representative of the other cycles and samples. Photographs of the (**b**) swollen state and shrunken states immediately after (**c**) convective heating and (**d**) photothermal heating with common scale bar (2 mm) shown in the left panel of (**c**). (**e**) Images from a thermal imaging camera immediately after photothermal heating, corresponding to (**d**). Note: Panels (**b**–**d**) were corrected to remove tick marks from a ruler on the edge of the photographs. An uncorrected version of these panels is presented in Figure S7.

Differences in the loading of BSA-GNRs in the crosshatched structures were readily discernible by eye, while the unloaded samples were colorless and transparent in the swollen state (Figure 3 and Figure S3) as a result of PAG. To quantify differences in the loading of BSA-GNRs, we refer to samples based on the extinction at the LSPR maximum, which is synonymous with the optical density (OD), of the colloidal BSA-GNR solution when measured in a glass cuvette with 1 cm path length; the actual OD of features within the printed structures was lower because they were much thinner, 3.6 mm in height. Four different loadings of BSA-GNRs were investigated, OD 2.5, 5, 10, and 20. As expected, increasing the loading of BSA-GNRs created a deeper purple hue in the sample. There was no indication of agglomeration or alignment of BSA-GNRs in the printed structures, which was confirmed by scanning electron microscopy of a cross-section from an OD 5 print (Figure S4). These images also show that the loading of BSA-GNRs was quite dilute, despite their strong extinction. The most concentrated sample, OD 20, was four times the concentration of OD 5 but still had a dilute volume or mass loading of BSA-GNRs. Therefore, we expected little to no agglomeration of BSA-GNRs in any of the samples because of their low loadings.

Compression testing was performed on bulk samples to compare the mechanical properties of an unloaded sample and an OD 10 sample, which was selected to represent an intermediate level of loading of GNRs (Figure S5). Cylindrical samples were prepared in molds using the same inks as for 3D printing and polymerized with the same UV light under conditions to mimic 3D printing. Both materials exhibited the mechanical properties of elastomers, but there were significant differences between them. At low strains below ≈0.05, both samples had similar stress vs. strain curves, above which the stress for the unloaded sample increased more rapidly with increasing strain. Consequently, the unloaded sample was mechanically tougher, although both samples fractured at strains near 0.65. These measurements show that GNRs had a significant effect on the mechanical properties of the PNIPAAm hydrogels. The volumetric loading of GNRs was quite dilute in all samples, so they were unlikely to directly influence the mechanical properties via shear thickening. Absorption of UV light by the GNRs would interfere with crosslinking and make the sample mechanically softer, which was reflected in the measurements, as the unloaded sample had a fracture strength of 39 kPa, while the OD 10 sample fractured at 22 kPa at a similar strain. The stress–strain curve for the loaded sample was smooth and consistent with a highly disordered microstructure, but there were several peaks in the stress–strain curve of the unloaded sample. Investigating the origin of those peaks was beyond the scope of this study. Copolymers or additives are often included in PNIPAAm for enhancing the 3D printability or mechanical toughness, and the materials in this work were softer than in most comparable studies [23,34,35]. It is also important to note that photopolymerization of a slab for mechanical testing here is different from a 3D-printed crosshatched structure because of the different geometry and fabrication processes, including how the sample was illuminated and the sequential nature of 3D printing. Furthermore, this 3D-printed mesh structure was a mechanical metamaterial, in which the mechanical properties depend on both the intrinsic properties of PNIPAAm and of the 3D-printed structure. Therefore, in significant ways, the mechanical properties of contiguous PNIPAAm slabs do not represent the total mechanical properties.

### 3.3. Photothermal Heating and Convective Heating

Incorporating GNRs makes photothermal heating of the 3D-printed PNIPAAm structures possible and induces shrinkage when the temperature is raised above the LCST. Cycles of photothermal heating and convective heating were compared for samples with different loadings of BSA-GNRs and an unloaded control sample by heating the sample above the LCST, driving collapse of PNIPAAm, and then allowing the sample to reswell prior to beginning the next cycle. During cycling, each sample was submerged in the minimum amount of water that fully covered it. For each sample loaded with GNRs, one cycle of convective heating and reswelling was conducted, followed by three cycles of photothermal heating and reswelling (Figure 3, Movies S3–S6). In comparison, the unloaded control sample was cycled through one round of convective heating, followed by illumination under the same conditions as for the loaded samples, but it did not undergo photothermal heating even over extended times (Figure S3, Movie S7). For reference, the LCST of the unloaded PNIPAAm hydrogel was 30.5 °C [56]. The BSA-GNRs were not expected to significantly affect the LCST, because they were not covalently crosslinked to PNIPAAm but were instead physically entrapped within the pores. In thermal imaging measurements during photothermal heating, collapse occurred consistently near 29 °C (Figure 3e). This was slightly below the referenced LCST, but thermal imaging was most sensitive to the surface of the sample, which in this instance was a thin layer of water on top of the PNIPAAm structure. This behavior was expected, because the layer of water transported heat away from the photothermally heated hydrogel. Under all conditions, the BSA-GNRs did not leach out of the PNIPAAm structure and remained within the 3D-printed piece, most notably during convective/photothermal cycling and after long-term storage. The 3D-printed structure retained a characteristic purple color, and the water bath was colorless. No leaching was observed during removal of the structure from the acetylated gelatin support matrix or after centrifuging or grinding bulk polymerized samples, conditions which were harsher than those experienced during 3D-printing or cycling (experiments not reported). Furthermore, the 3D-printed structures responded consistently (i.e., speed and extent of actuation) during multiple rounds of photothermal cycling. A total of four cycles are reported here, comparable with related reports [18,20,35,52]. This thermal responsiveness is an intrinsic property of PNIPAAm, and the BSA-GNRs that enable photothermal heating were well stabilized and trapped within the PNIPAAm pores, which prevented them from diffusing into the water bath.

An LED with a peak wavelength of 850 nm was chosen for photothermal heating in this work to overlap with the LSPR. Photothermal heating is fast and localized, which enables actuation of the 3D-printed structures in water baths at temperatures well below the LCST (Movie S8). Several measurements were taken across the four rounds of actuation on the OD 2.5 and 10 samples to analyze the dynamics of the shrinkage and reswelling processes (Figure S6). The speeds of actuation for both convective heating and all rounds of photothermal heating were similar, but convective heating had a slower onset to shrinkage because the entire water bath first needed to heat above the LCST of the 3D-printed hydrogel. Similarly, the reswelling process after photothermal heating was more rapid, because the water bath remained near room temperature throughout the entire process. Reswelling after convective heating was slower, but there was a greater extent of reswelling, because adding ice to cool the water bath brought it below room temperature. The extent of shrinkage was consistent across both modes of actuation and for all loadings of GNRs.

### 3.4. Effect of Loading of GNRs

There was a subtle effect of the loading of GNRs, where higher loadings slightly increased the extent of shrinkage (Figure 3). Since this effect was observed for both modes of heating, we attribute it to morphological differences in the hydrogels caused by absorption of UV light by the GNRs during photopolymerization. We propose that higher loadings of GNRs more strongly interfere with polymerization, resulting in a more loosely connected network that can shrink to a smaller size. Under convective heating, the unloaded control sample shrunk to only ≈45% of its initial area, in comparison with the 20–30% achieved by the loaded samples (Figure S3, Movie S7). The higher average elastic modulus of the unloaded sample (Figure S5) was consistent with a greater extent of crosslinking than in a more loosely connected network with GNR incorporation. As expected, the unloaded control sample did not undergo photothermal heating, because there was minimal extinction at 850 nm without the GNRs (Figure S3). Logically, there must be a range of concentrations over which the efficacy of photothermal heating correlated with the loading of GNRs, but we did not observe it. This regime for the onset of photothermal heating therefore occurred at concentrations of GNRs below OD 2.5, even though that sample appeared nearly transparent by eye.

As already noted, higher loadings of GNRs provided slightly more collapse of the structures, but the convective and thermal cycling behaviors were largely insensitive to the level of loading of GNRs. Predicting the trend of the behavior across different loadings is non-trivial. Therefore, we briefly discuss several effects that could in principle contribute to different behaviors but would be challenging to disentangle. For example, higher loadings of GNRs are expected to inhibit photopolymerization during 3D printing, but they will also absorb more light during photothermal heating. Increasing the loading of GNRs would be expected to cause faster actuation up to a limit, where most of the light would be absorbed on the surface of the sample, thus reducing the intensity of light penetrating into the sample. This, together with reduced photopolymerization during fabrication, suggests there is an upper limit for the GNR concentration, above which the performance is diminished, but we did not observe this even for the deeply pigmented OD 20 sample.

### 3.5. Kinetics of Collapse and Reswelling

Movies S3–S7 of convective heating followed by photothermal heating allow for more detailed observations and discussion. Convective heating was conducted by placing the sample in a room-temperature water bath, followed by rapidly heating the bath’s temperature above the LCST, to ≈35 °C. The 3D-printed structures shrunk over a period of 5–8 min. For thermoresponsive centimeter-scale structures such as these, actuation typically occurs over several minutes [26], which is consistent with these results. Smaller microstructures, in contrast, can collapse in less than 1 s [18]. After 3D-printing and during sample storage, the structure can slightly and slowly swell, with the edge length of the print increasing by ≈1 mm. To mitigate this initial swelling beyond the 3D-printed dimensions, a first cycle of convective heating was performed but not reported.

Three cycles of photothermal heating were conducted to more thoroughly investigate this mode of actuation that is only achieved by adding GNRs. For all loadings of GNRs and independent of the extent of shrinkage, the shrinkage process was nonuniform because the LED illumination was noniform. During photothermal heating, the structures shrank over 5–10 min, similar to convective heating. Photothermal heating could be further accelerated by using a laser, but we chose an LED because it is simpler to implement and mitigates safety challenges of working with lasers, particularly with light outside of the visible spectrum.

Measurements of the area taken over time for the OD 2.5 and 10 samples (Figures S8 and S9) were fit by an exponential function with a constant offset, $Ae^{-kt}+c$, where *c* represents the area of the collapsed hydrogel. Values of the rate constant *k* (Tables S2 and S3) were in the range of 1–3 min^−1^ for convective heating and three cycles of photothermal heating for both samples. This suggests the rate of shrinkage was intrinsically limited by the collapse behavior of PNIPAAm above the LCST. We did not observe significant differences in the rates of collapse and reswelling for the OD 2.5 and OD 10 samples. Furthermore, the experiments were conducted with neither quantitative control of the heating and cooling rates of the water bath nor controlled placement of the sample as it floated on water above the LED.

### 3.6. Buckling during Reswelling

**Figure 4:**
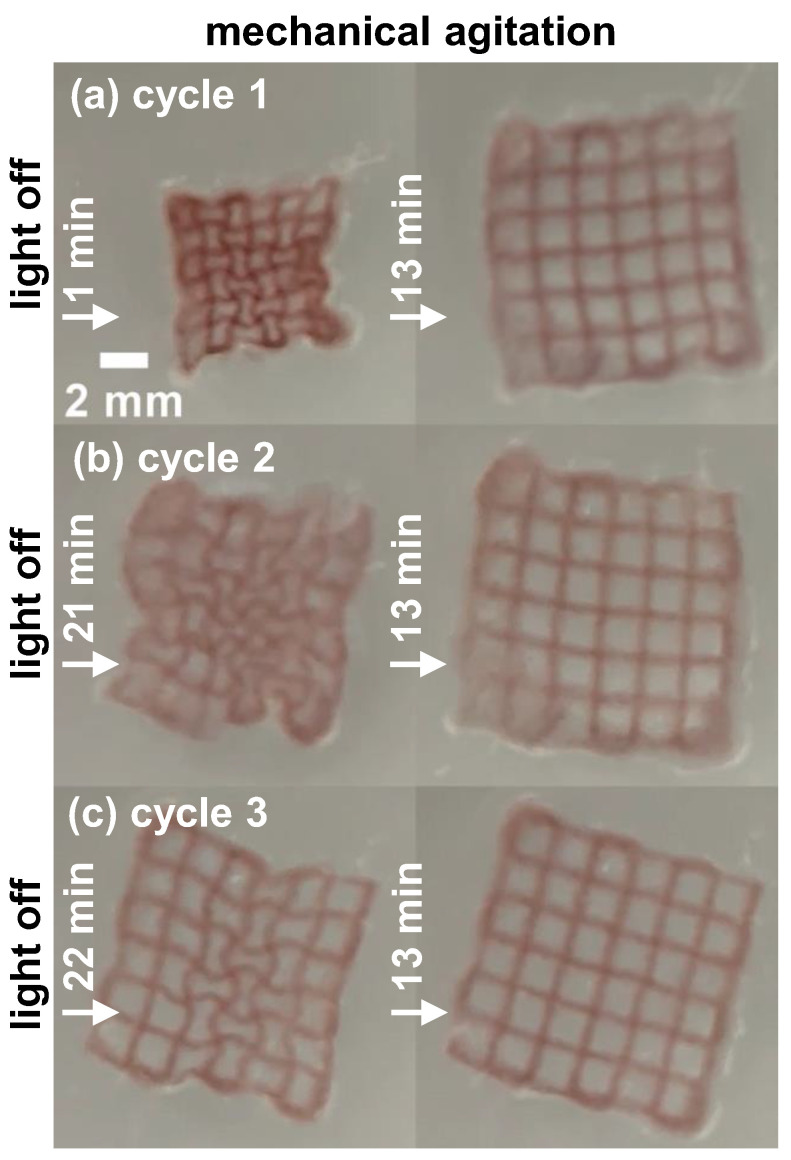
(**a**–**c**) Photographs of an OD 20 sample, which underwent inward buckling during reswelling after successive cycles of photothermal heating. For each cycle, the left image shows an image of a buckled state, and the right image is after complete unbuckling and reswelling. The images are snapshots taken from Movie S6.

Two samples, OD 5 and OD 20, consistently exhibited an unexpected behavior during reswelling after photothermal heating, where walls of the square unit cells converted into interlocking dog-bone like shapes (Figure 4 and Figure S10), which is known as a sinusoidal ligament pattern [67]. Such two-dimensional meshes with curved or zigzag edges can exhibit auxetic behaviors, meaning they have an effective negative Poisson ratio, by buckling during compression and unbuckling during expansion. Auxetic behaviors are most commonly investigated in elastomers under external forces [68,69,70], but similar behaviors have been observed in thermoresponsive systems [71,72]. Here, buckling was observed during reswelling only after photothermal heating. Therefore, the cause must be distinct to photothermal heating. For example, because of the relatively small size of the LED compared with the 3D-printed structure, the corners of the structure can taper (i.e., have their angles reduced below 90°) during photothermal heating, which could serve to template the edges of an inwardly buckled unit cell. The sequence of 3D printing also appeared to influence the buckling pattern, where the slightly rounded turns on the edge of the structure of 90° followed by another 90° corresponded to segments buckled outward, and the straight segments on the edge of the structure buckled inward. Adding a few drops of water mechanically agitated the structures, quickly driving unbuckling. Why only some samples exhibited this behavior after photothermal heating was not clear, but minor variations in many variables could play a role, such as the 3D printing process, extent and spatial distribution of photopolymerization, and preparation and properties of the matrix for embedded 3D printing.

## 4. Conclusions

PNIPAAm-GNR composites were created through photoinitiated PAG by embedded 3D printing in an acetylated gelatin hydrogel matrix, which was designed to melt below the LCST of PNIPAAm. For low loadings of GNRs, the structures were highly transparent, while they became nearly opaque at high loadings of GNRs. The GNRs remained in the structure even after several cycles of convective and photothermal heating and reswelling, and structures 3D printed with a range of GNR loadings exhibited similar actuation behaviors. The observation of buckling during reswelling after photothermal heating of the OD 5 and OD 20 samples points toward an opportunity to purposefully design hydrogel structures to incorporate snapping or auxetic behaviors if the variables in this complex system are further optimized [70,73,74,75]. While PNIPAAm is chiefly a model system, embedding plasmonic NPs and photothermal heating could be extended to related biocompatible 3D-printed hydrogel structures.

The consistency of shrinkage during photothermal heating is remarkable because the loading of GNRs affected both photopolymerization of the hydrogel and extinction of light during photothermal heating. Moreover, the OD 2.5 sample appeared only faintly colored by the eye, yet it exhibited excellent performance. This suggests an opportunity for developing multifunctional systems, where other additives could introduce novel behaviors in visible wavelengths, while not interfering with NIR photothermal heating. Furthermore, advancements in multimaterial 3D printing provide the means to 3D print heterostructures that could exhibit novel responses under different colors of light (e.g., using GNRs with different aspect ratios). Precisely this functionality is envisioned for future applications in tissue engineering and microsurgery. The ability to collapse and reswell 3D-printed hydrogel meshes with customized geometries through external triggering will allow for wrapping, transporting, and releasing selected cells and tissue samples on demand [76,77,78].

## Data Availability

Data are contained within the article and Supplementary Materials.

## Supplementary Materials

The following supporting information can be downloaded at https://www.mdpi.com/article/10.3390/polym16142032/s1. Table S1: LSPR peak position and full width at half maximum (FWHM) from extinction spectra of CTAB- and BSA-functionalized GNRs, and *ζ*-potential measurements. Figure S1: ^1^H NMR of PNIPAAm macromer for quantifying the molar concentration of photocrosslinkable DMMIAAm groups. Figure S2: ^1^H NMR of acetylated gelatin and native gelatin. The highlighted region **a** indicates the presence of methyl protons from acetyl groups attached to the gelatin backbone, and region **b** shows modification of a lysine moiety within the gelatin structure. The observed changes upon acetylation agree with literature values. From C. Claaßen, M. H. Claaßen, V. Truffault, L. Sewald, G. E. M. Tovar, K. Borchers, A. Southan, *Biomacromolecules* **2018**, *19*, 42. Figure S3: Images of (a) convective and (b) photothermal heating of an unloaded, 3D-printed control sample. An orange background is used to enhance the contrast in the highly transparent, swollen state. Figure S4: SEM images from backscattered electrons of the cross-section of a 3D-printed OD 5 PNIPAAm-GNR structure at (a) low magnification and (b) high magnification, which show that the GNRs were well dispersed, with minimal agglomeration and no apparent orientation. Figure S5: Unconfined compression testing results comparing an unloaded and loaded (OD 10) sample, plotting true compressive stress vs. compressive strain, −Δ*L*/*L*_0_. The samples were polymerized in bulk in a cylindrical mold, 5 mm in diameter with a height of 4 mm. A compressive load of 5 N was applied for these measurements at 25 °C and with a strain rate of 1 mm min^−1^. Figure S6: Plots of collapse and reswelling over time for (a) OD 2.5 and (b) OD 10 samples during one cycle of convective heating followed by three cycles of photothermal heating (C1, C2, C3). Neither the time of illumination (4.2–5.2 min) nor the reswelling time were fixed. The final point corresponds to the last frame of that cycle, before starting the next cycle. Figure S7: Uncorrected version of Figure 3b–d of the main text showing the tick marks from a ruler in the edges of some photographs. Figure S8: Fitting of collapse behavior for the OD 2.5 sample for (a) convective cycling and (b–d) three sequential cycles of photothermal heating. The area of the structure was measured over discrete time points during the collapse and then fitted to an exponential decay function of best fit. Time *t* = 0 is offset to the first point measured where shrinkage begins. Table S2: Parameters from fitting to the exponential decay function over the convective and photothermal cycles for the OD 2.5 sample. The exponential decay function was $A=A_{1}e^{-k_{1}t}+y_{0}$. Figure S9: Fitting of collapse behavior for the OD 10 sample for (a) convective cycling and (b–d) three sequential cycles of photothermal heating. The area of the structure was measured over discrete time points during the collapse and then fitted to an exponential decay function of best fit. Time *t* = 0 is offset to the first point measured where shrinkage begins. Table S3: Parameters from fitting to the exponential decay function over the convective and photothermal cycles for the OD 10 sample. The exponential decay function was $A=A_{1}e^{-k_{1}t}+y_{0}$. Figure S10: (a–c) Photographs of the OD 5 sample, which underwent inward buckling during reswelling after successive cycles of photothermal heating. For each cycle, the left image shows a buckled state at a time selected for clarity, and the right image is after complete unbuckling and reswelling. The snapshots are taken from Movie S4, Supplementary Materials. Figure S11: Side-view photograph of an OD 20 sample after removal of the microgel support structure made of acetylated gelatin. Figure S12: Photograph of the setup for convective heating of 3D-printed hydrogels. A small enclosure was constructed from stacked glass slides to prevent sample movement and keep the sample within the camera’s field of view. The light in the front provides additional contrast for taking photographs of the samples with low loadings of GNRs. Figure S13: Photographs of the setup for photothermal heating. A glass Petri dish was filled with ultrapure water until the sample was completely submerged, and then it was placed below an 850 nm LED. A thermal imaging camera was placed further away to record the changes in temperature as the sample underwent photothermal heating. Movie S1: Printing process, real-time. Movie S2: Removal of acetylated gelatin support after post-cure, 5× speed. Movie S3: Convective and photothermal heating of OD 2.5 hydrogel. Movie S4: Convective and photothermal heating of OD 5 hydrogel. Movie S5: Convective and photothermal heating of OD 10 hydrogel. Movie S6: Convective and photothermal heating of OD 20 hydrogel. Movie S7: Convective and photothermal heating of unloaded hydrogel. Movie S8: Demonstration of photothermal heating at low temperature, OD 20 hydrogel.
