# Supplementary material for: 3D-Printed Hydrogels as Photothermal Actuators
Source: Polymers (Basel). 2024 Jul 17;16(14):2032. doi: 10.3390/polym16142032 (PMC11281285; doi:10.3390/polym16142032)
Supplement: Supplementary file 1 [file polymers-16-02032-s001.zip › polymers-3069996-supplementary.pdf]

Supplementary Materials

3D-Printed Hydrogels as Photothermal Actuators

Melanie M. Ghelardini [1], Martin Geisler [2], Niclas Weigel [2], Jameson P. Hankwitz [1], Nicolas Hauck [2], Jonas Schubert [2], Andreas Fery [2,3], Joseph B. Tracy [1,*] and Julian Thiele [2,4,*]

[1]Department of Materials Science and Engineering, North Carolina State University, Raleigh, North Carolina 27695, United States

[2]Leibniz Institute of Polymer Research Dresden, Institute of Physical Chemistry and Polymer Physics, 01069 Dresden, Germany

[3]Institute of Physical Chemistry and Polymer Physics, Technische Universität Dresden, 01062 Dresden, Germany

[4]Institute of Chemistry, Otto von Guericke University Magdeburg, Universitätsplatz 2, 39106 Magdeburg, Germany

Email: julian.thiele@ovgu.de, jbtracy@ncsu.edu

Additional Information for Ink and Printing Components:

Table S1. LSPR peak position and full width at half maximum (FWHM) from extinction spectra of CTAB- and BSA-functionalized GNRs, and ζ-potential measurements.

	**LSPR Peak (nm)**	**FWHM (nm)**	**ζ-Potential (mV)**
**CTAB-GNRs**	802	120	45.3
**BSA-GNRs**	792	123	-28.1

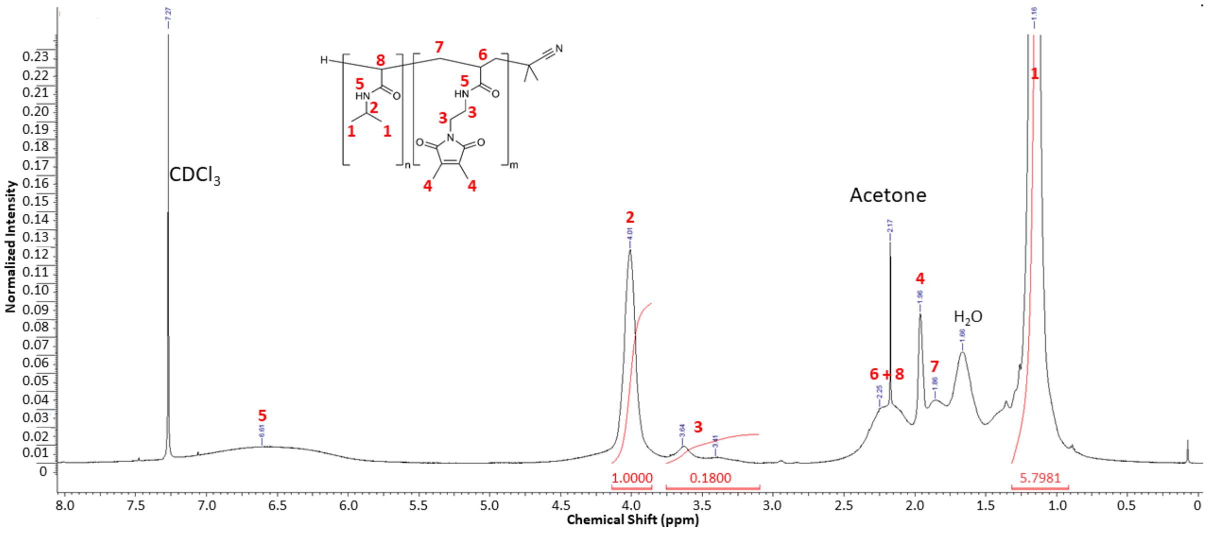

Figure S1. ^{1}H NMR of PNIPAAm macromer for quantifying the molar concentration of photocrosslinkable DMMIAAm groups.

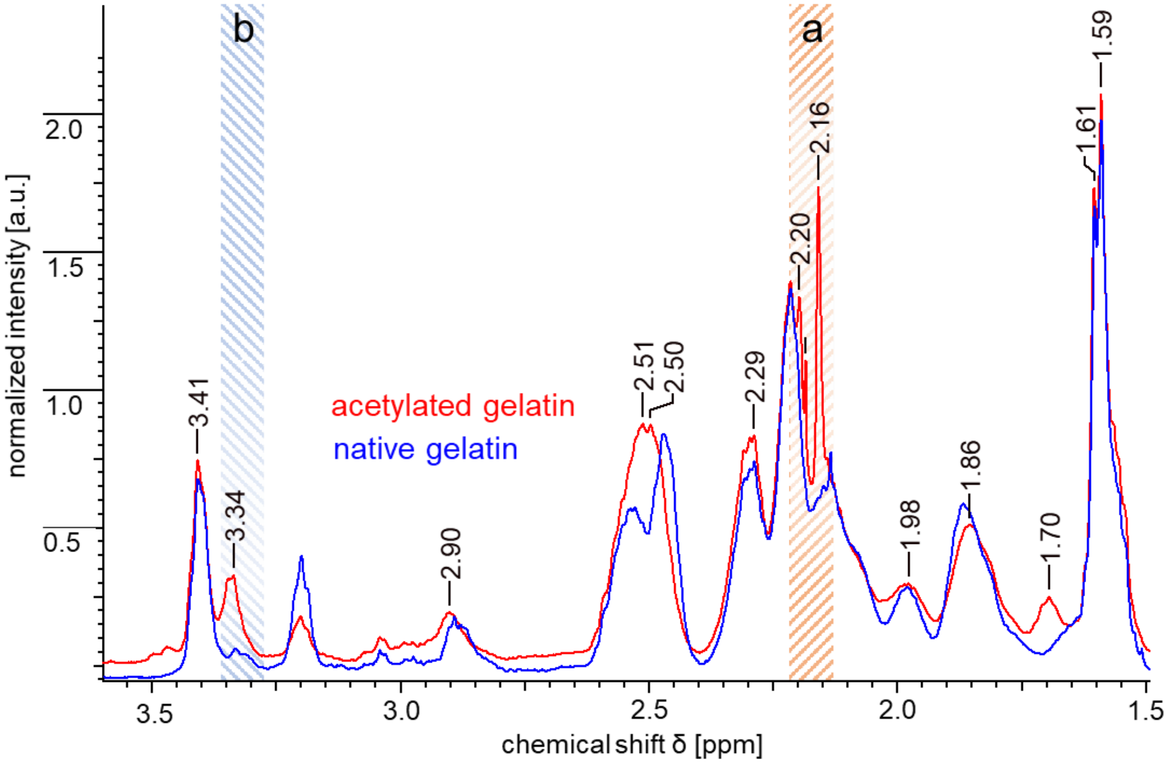

Figure S2. ^{1}H NMR of acetylated gelatin and native gelatin. The highlighted region **a** indicates the presence of methyl protons from acetyl groups attached to the gelatin backbone, and region **b** shows modification of a lysine moiety within the gelatin structure. The observed changes upon acetylation agree with literature values. From C. Claaßen, M. H. Claaßen, V. Truffault, L. Sewald, G. E. M. Tovar, K. Borchers, A. Southan, *Biomacromolecules* **2018**, *19*, 42.

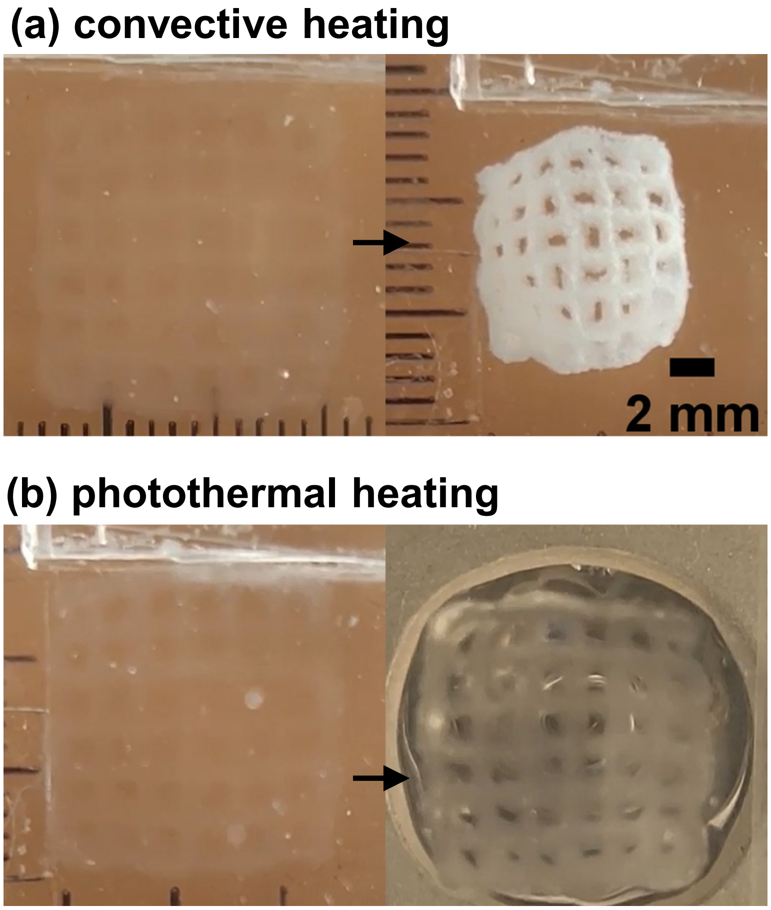

Figure S3. Images of (a) convective and (b) photothermal heating of an unloaded, 3D-printed control sample. An orange background is used to enhance the contrast in the highly transparent, swollen state.

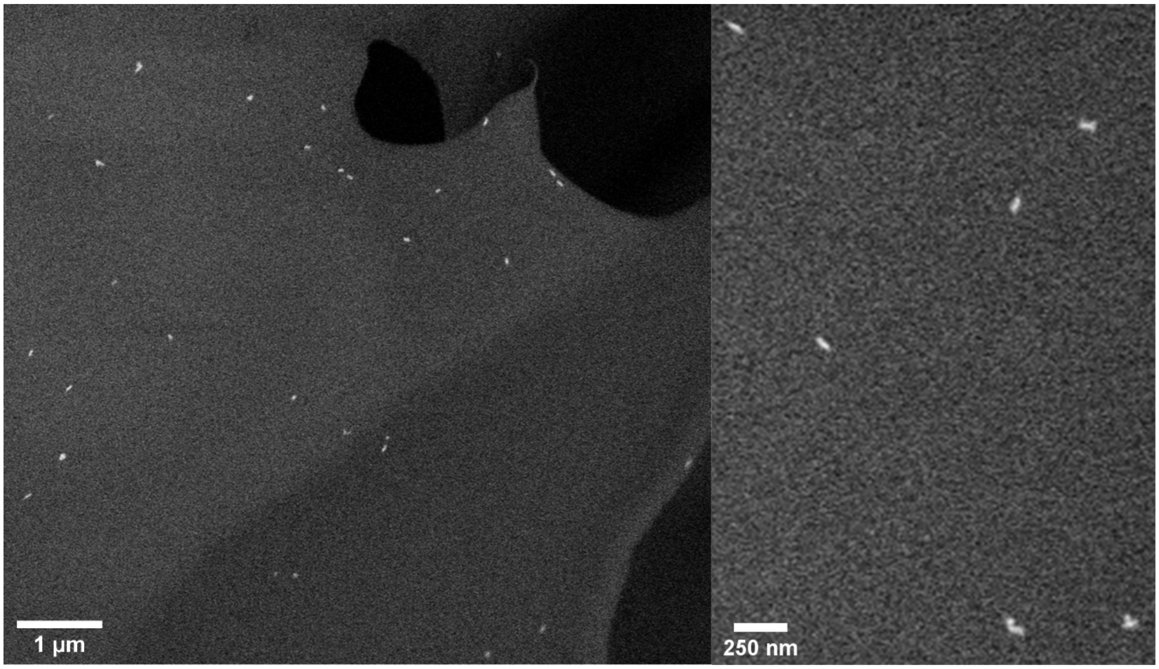

Figure S4. SEM images from backscattered electrons of the cross-section of a 3D-printed OD 5 PNIPAAm-GNR structure at (a) low magnification and (b) high magnification, which show that the GNRs were well dispersed, with minimal agglomeration and no apparent orientation.

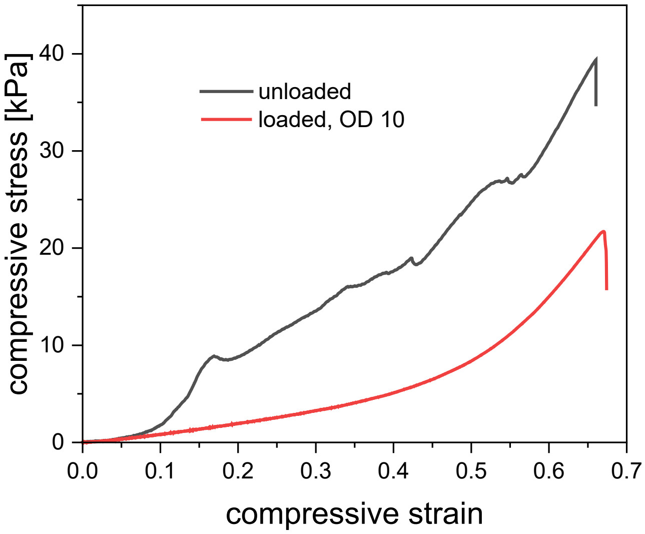

Figure S5. Unconfined compression testing results comparing an unloaded and loaded (OD 10) sample, plotting true compressive stress vs. compressive strain, $-\Delta L/L_0$. The samples were polymerized in bulk in a cylindrical mold, 5 mm in diameter with a height of 4 mm. A compressive load of 5 N was applied for these measurements at 25 °C and with a strain rate of 1 mm min^{-1}.

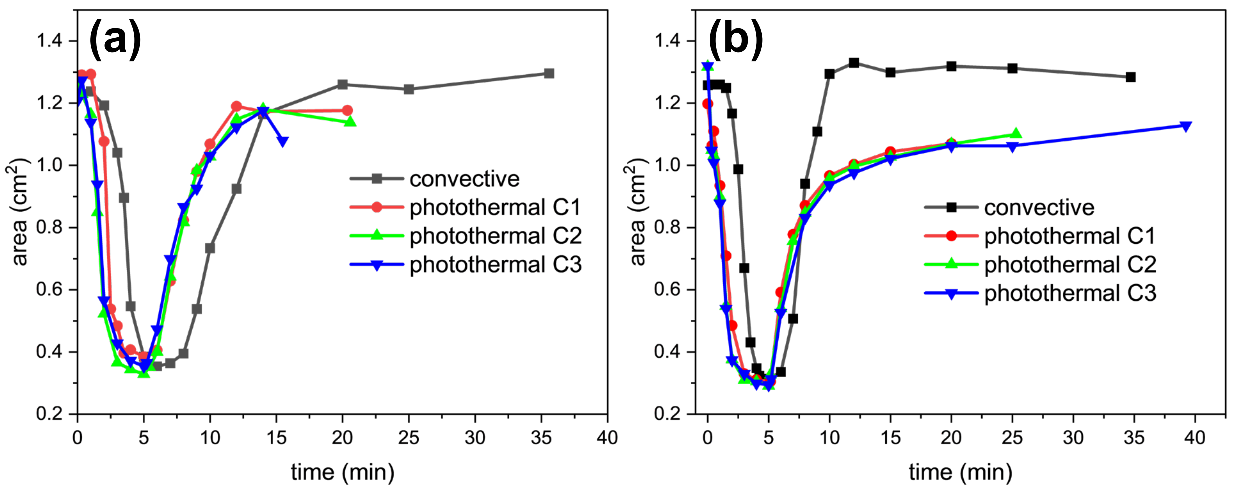

Figure S6. Plots of collapse and reswelling over time for (a) OD 2.5 and (b) OD 10 samples during one cycle of convective heating followed by three cycles of photothermal heating (C1, C2, C3). Neither the time of illumination (4.2 – 5.2 min) nor the reswelling time were fixed. The final point corresponds to the last frame of that cycle, before starting the next cycle.

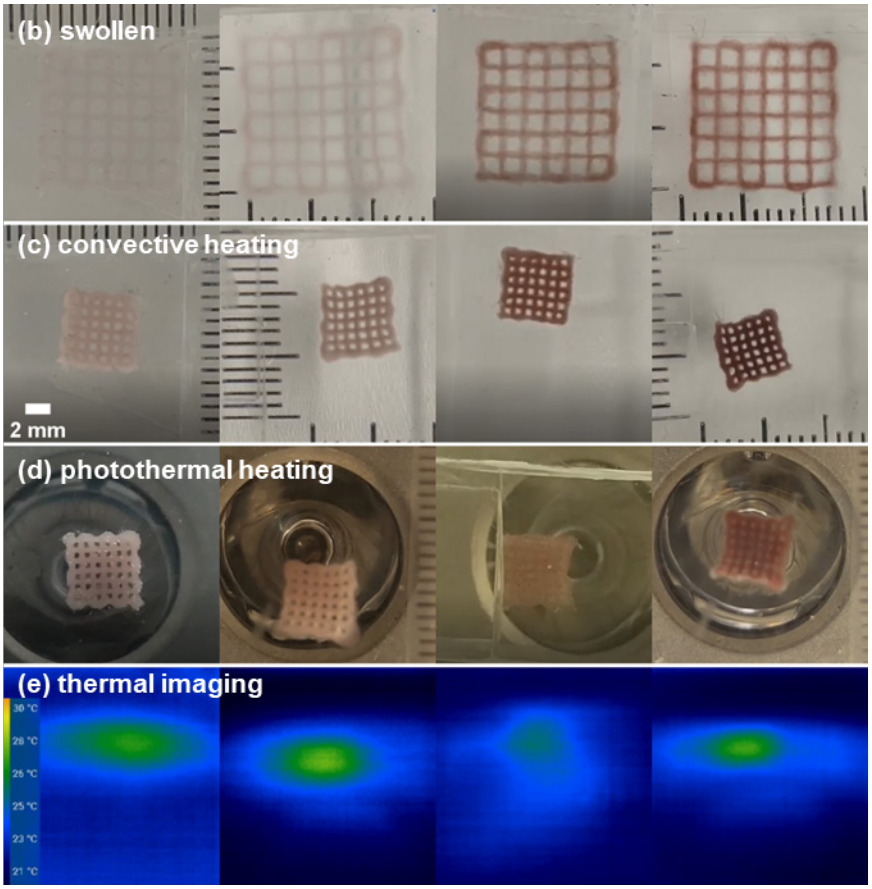

Figure S7. Uncorrected version of Figure 3b-d of the main text showing the tick marks from a ruler in the edges of some photographs.

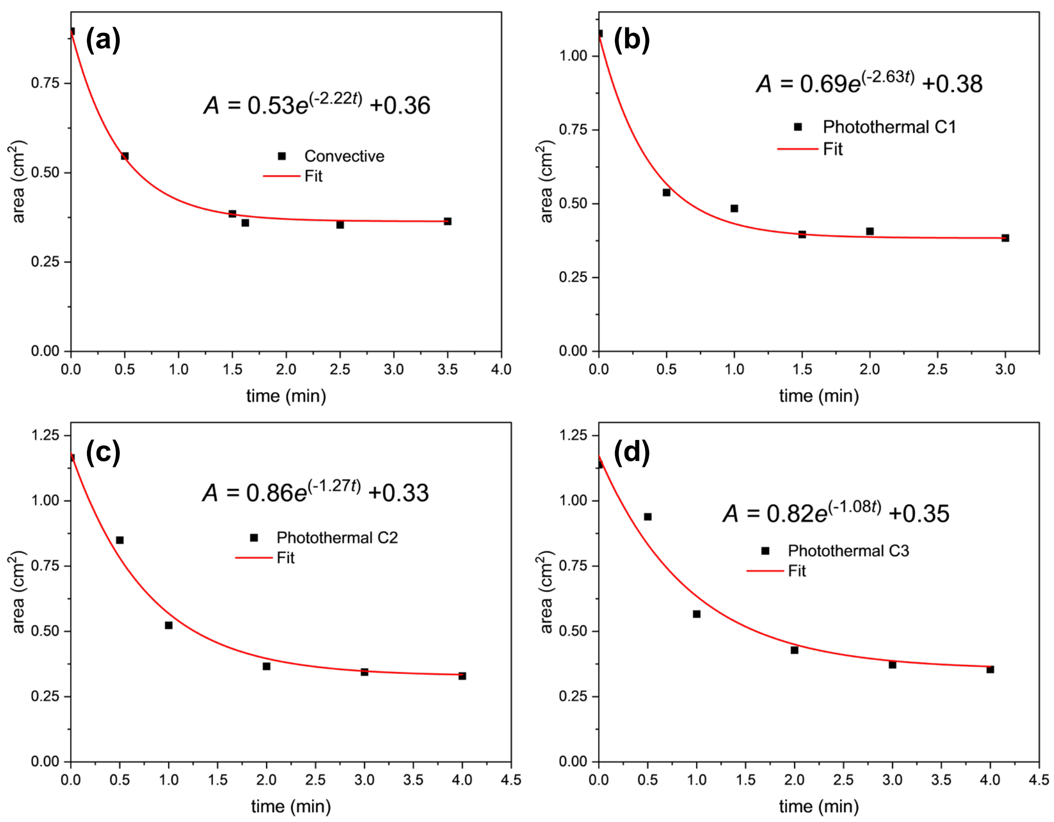

Figure S8. Fitting of collapse behavior for the OD 2.5 sample for (a) convective cycling and (b-d) three sequential cycles of photothermal heating. The area of the structure was measured over discrete time points during the collapse and then fitted to an exponential decay function of best fit. Time $t = 0$ is offset to the first point measured where shrinkage begins.

Table S2. Parameters from fitting to the exponential decay function over the convective and photothermal cycles for the OD 2.5 sample. The exponential decay function was $A = A_1 e^{-k_1 t} + y_0$.

Cycle	A_1 (cm^2)	k_1 (min^{-1})	y_0 (cm^2)
Convective	0.53 ± 0.01	2.22 ± 0.15	0.36
Photothermal 1	0.69 ± 0.03	2.63 ± 0.28	0.38
Photothermal 2	0.86 ± 0.04	1.27 ± 0.14	0.33
Photothermal 3	0.82 ± 0.06	1.08 ± 0.18	0.35

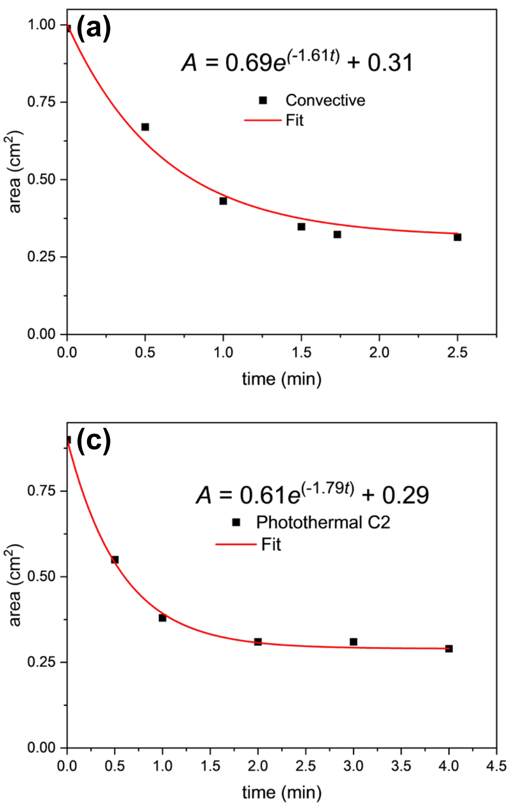

Figure S9. Fitting of collapse behavior for the OD 10 sample for (a) convective cycling and (b-d) three sequential cycles of photothermal heating. The area of the structure was measured over discrete time points during the collapse and then fitted to an exponential decay function of best fit. Time $t = 0$ is offset to the first point measured where shrinkage begins.

Table S3. Parameters from fitting to the exponential decay function over the convective and photothermal cycles for the OD 10 sample. The exponential decay function was $A = A_1 e^{-k_1 t} + y_0$.

Cycle	A_1 (cm^2)	k_1 (min^{-1})	y_0 (cm^2)
Convective	0.69 ± 0.03	1.61 ± 0.16	0.31
Photothermal 1	0.65 ± 0.03	1.19 ± 0.13	0.31
Photothermal 2	0.61 ± 0.01	1.79 ± 0.06	0.29
Photothermal 3	0.58 ± 0.01	1.82 ± 0.10	0.29

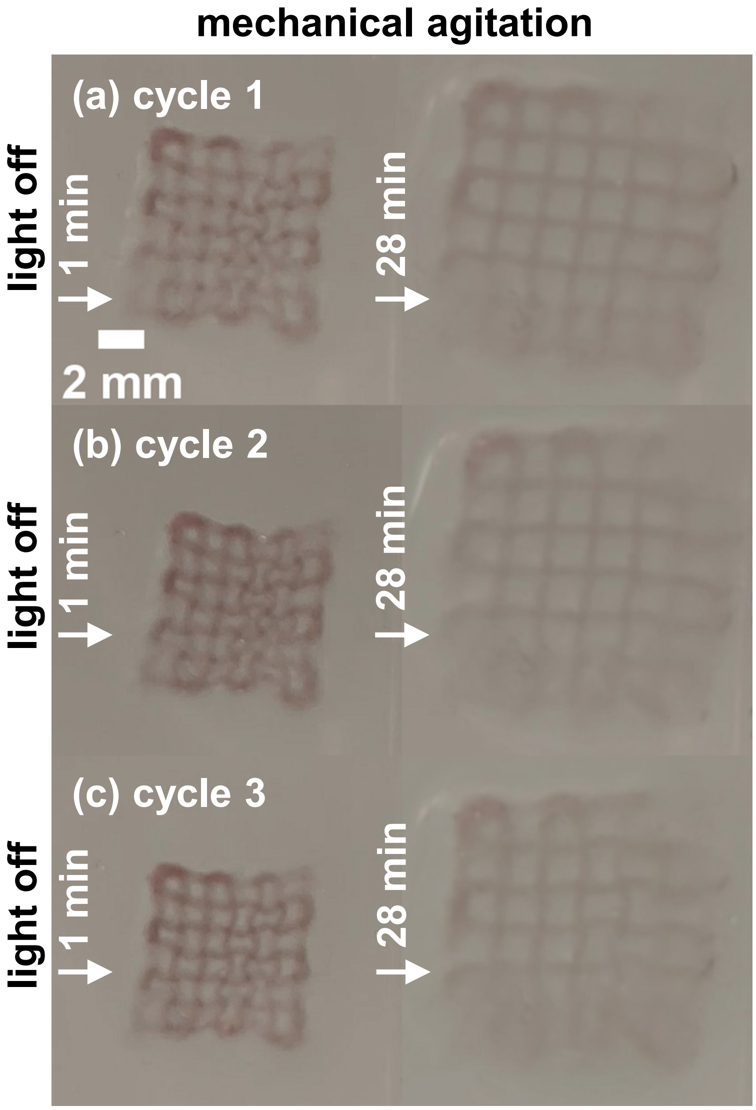

Figure S10. (a-c) Photographs of the OD 5 sample, which underwent inward buckling during reswelling after successive cycles of photothermal heating. For each cycle, the left image shows a buckled state at a time selected for clarity, and the right image is after complete unbuckling and reswelling. The snapshots are taken from Movie S4, Supplementary Materials.

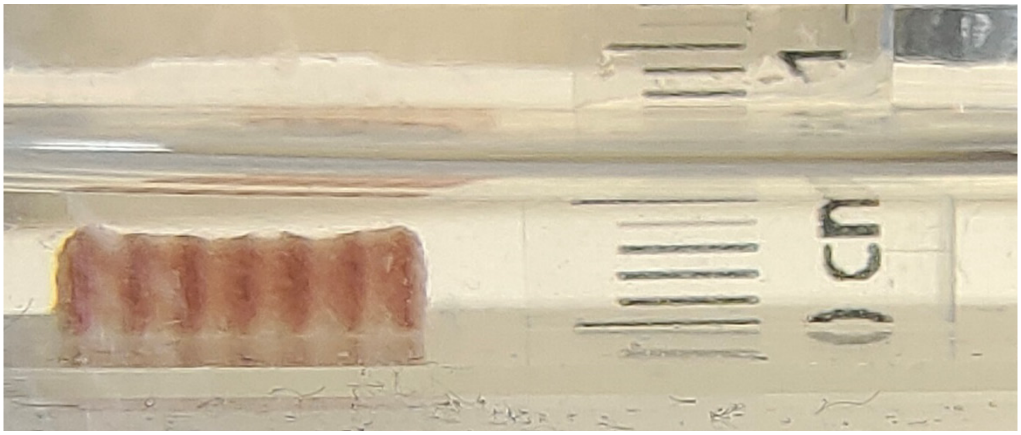

Figure S11. Side-view photograph of an OD 20 sample after removal of the microgel support structure made of acetylated gelatin.

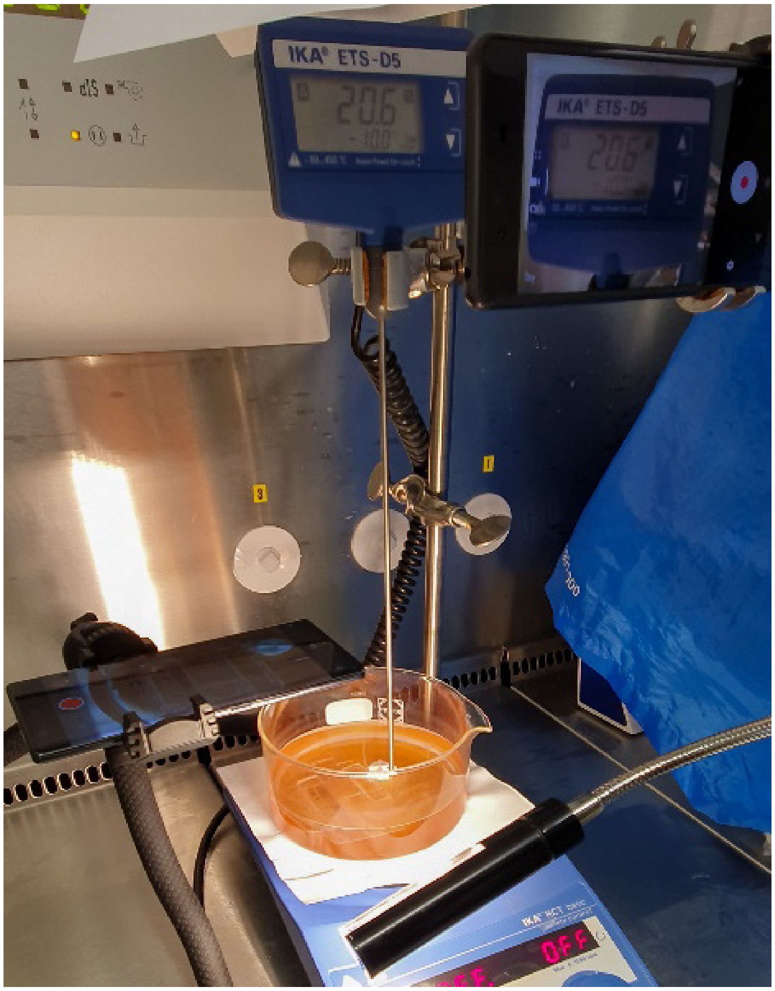

Figure S12. Photograph of the setup for convective heating of 3D-printed hydrogels. A small enclosure was constructed from stacked glass slides to prevent sample movement and keep the sample within the camera's field of view. The light in the front provides additional contrast for taking photographs of the samples with low loadings of GNRs.

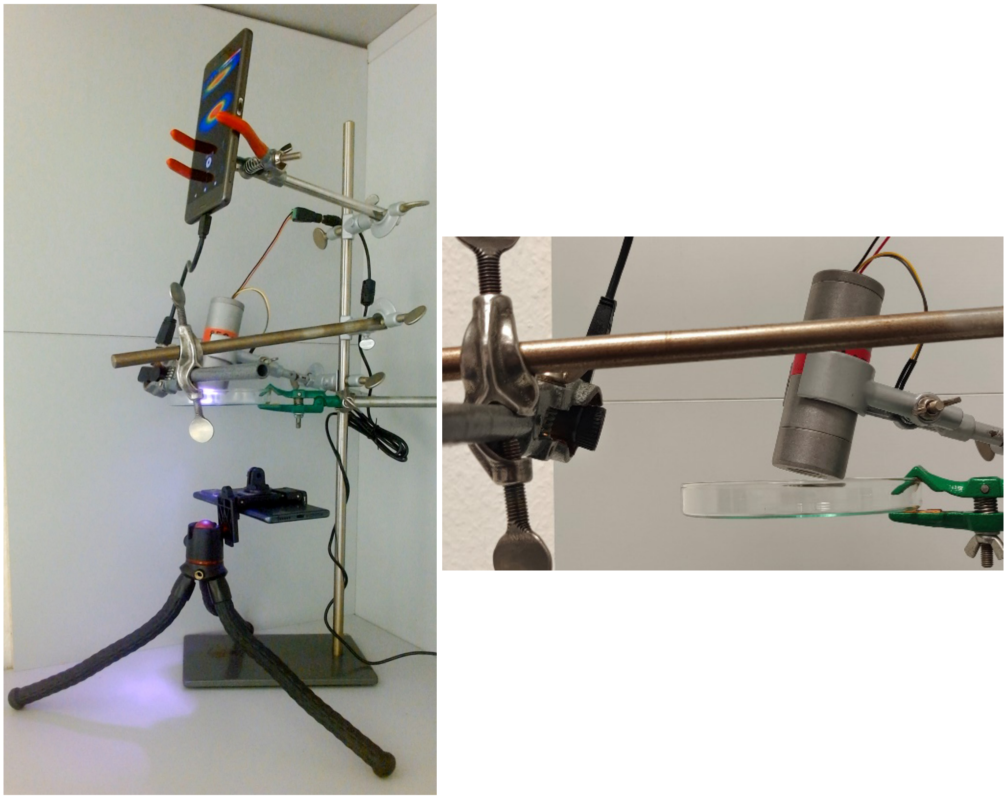

Figure S13. Photographs of the setup for photothermal heating. A glass Petri dish was filled with ultrapure water until the sample was completely submerged, and then it was placed below an 850 nm LED. A thermal imaging camera was placed further away to record the changes in temperature as the sample underwent photothermal heating.
